# Unusually high SO_2_ emissions and plume height from Piton de la Fournaise volcano during the April 2020 eruption

**DOI:** 10.1007/s00445-023-01628-1

**Published:** 2023-03-08

**Authors:** C. Hayer, M. Burton, V. Ferrazzini, B. Esse, A. Di Muro

**Affiliations:** 1 COMET, Department of Earth and Environmental Sciences, The University of Manchester, Manchester, M13 9PL UK; 2 Institut de Physique du Globe de Paris, Université de Paris, CNRS, 75005 Paris, France; 3 Institut de Physique du Globe de Paris, Observatoire Volcanologique du Piton de La Fournaise, 97418 La Plaine Des Cafres, France

**Keywords:** Gas emissions, Volcano seismicity, Remote sensing, TROPOMI, PlumeTraj

## Abstract

Piton de la Fournaise volcano, La Réunion, France, erupted between the 2 and 6 April 2020, one of a series of eruptive phases which occur typically two or three times per year. Here, we use back trajectory analysis of satellite data from the TROPOMI instrument to determine that gas emissions during the June 2020 eruption were of unusually high intensity and altitude, producing 34.9 ± 17.4 kt of SO_2_ and plume heights up to 5 km a.s.l. The early stages of the eruption (2–4 April 2020) were characterised by relatively low SO_2_ emission rates despite strong low frequency tremor (LFT); the latter phase followed an increase in intensity and explosivity in the early hours of 5 April 2020. This period included lava fountaining, significantly increased SO_2_ emission rates, increased high frequency tremor (HFT) and decreased LFT. Using the PlumeTraj back trajectory analysis toolkit, we found the peak SO_2_ emission rate was 284 ± 130 kg/s on the 6 April. The plume altitude peaked at ~ 5 km a.s.l. on 5 April, in the hours following a sudden increase in explosivity, producing one of the tallest eruption columns recorded at Piton de la Fournaise. PlumeTraj allowed us to discriminate each day’s SO_2_, which otherwise would have led to a mass overestimate due to the plumes remaining visible for more than 24 h. The eruption exhibited a remarkable decoupling and anti-correlation between the intensity of the LFT signal and that of the magma and gas emission rates. LFT intensity peaked during the first phase with low magma and SO_2_ emissions, but quickly decreased during the second phase, replaced by unusually strong HFT. We conclude that the observation of strong HFT is associated with higher intensity of eruption, degassing, and greater height of neutral buoyancy of the plume, which may provide an alert to the presence of greater hazards produced by higher intensity eruptive activity. This might be particularly useful when direct visual observation is prevented by meteorological conditions. This eruption shows the importance of combining multiple data sets when monitoring volcanoes. Combining gas and seismic data sets allowed for a much more accurate assessment of the eruption than either could have done alone.

**Supplementary Information:**

The online version contains supplementary material available at 10.1007/s00445-023-01628-1.

## Introduction

**Fig. 1:**
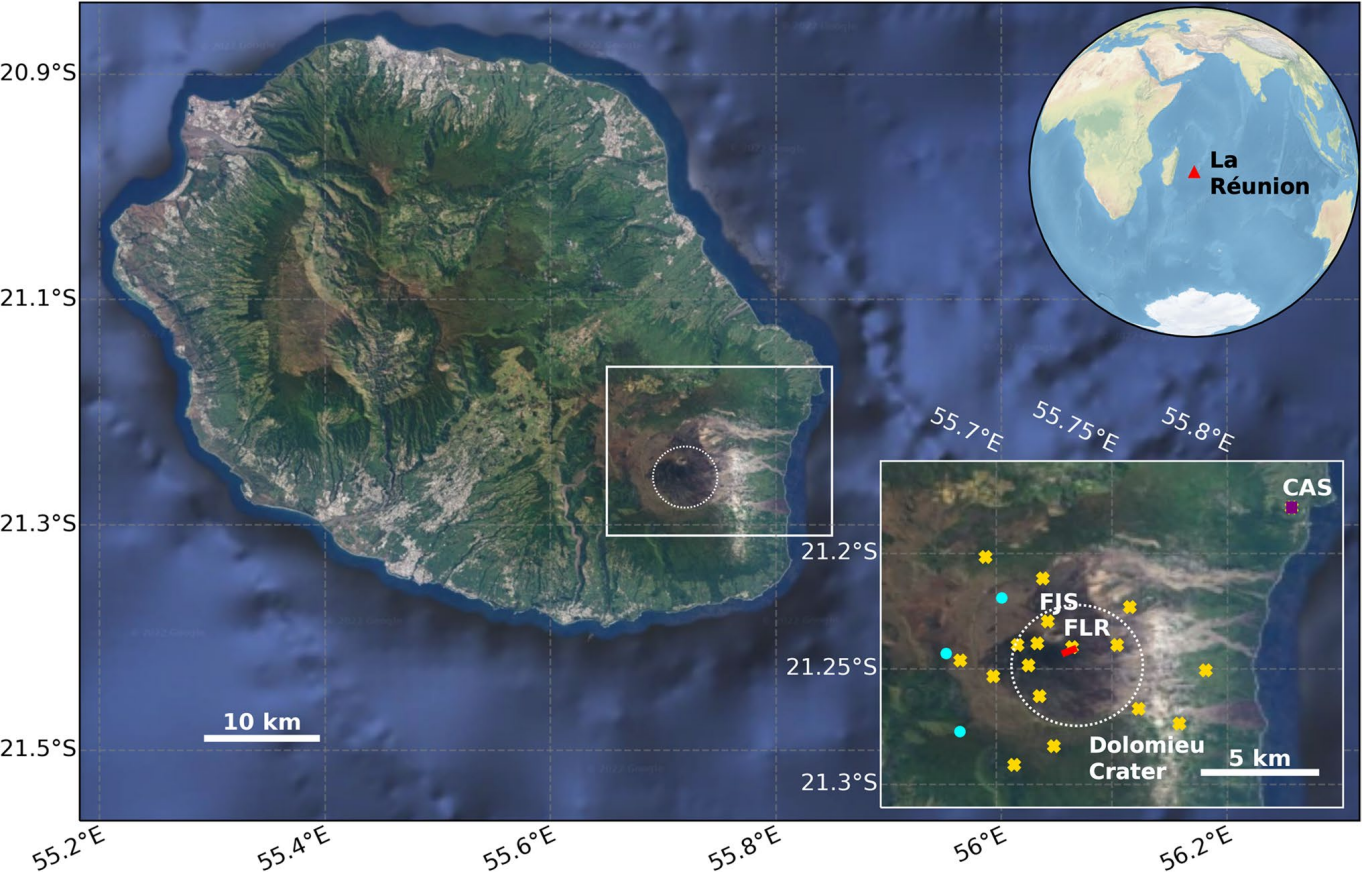
Main: map of La Réunion (white square denotes area shown in bottom inset); inset top: global location of La Réunion Island; inset bottom: region over Piton de la Fournaise, with monitoring stations denoted (purple square = visible camera, blue circles = MAX-DOAS, yellow crosses = broadband seismometers). Red line = eruptive fissure. Stations discussed within this work are noted. Main & bottom inset maps data: Google, ©2022 Landsat/Copernicus

Explosive eruptions with limited precursory signals are highly dangerous events, especially when the surrounding regions are inhabited. Basaltic volcanoes usually erupt under an effusive regime, so explosive events at these volcanoes, such as those seen at Mt. Etna, Italy (Coltelli et al. 1998; Houghton et al. 2004), and Masaya, Nicaragua (Bamber et al. 2020), can then be particularly dangerous as they are unexpected and often unpredictable. Piton de la Fournaise is a basaltic shield volcano located on the island of La Réunion, approximately 800 km off the east coast of Madagascar (Fig. 1) and is an example of intraplate volcanism, similar to the volcanoes of Hawai’i (Staudacher et al. 2016). Piton de la Fournaise is one of the most active basaltic volcanoes in the world, with an average of 2–3 eruptions per year, and occasional extended periods of quiescence lasting 1–6 years (Roult et al. 2012; Peltier et al. 2021). The most recent quiescent period ended in 2014 (Peltier et al. 2016; Boudoire et al. 2017).

Gas emissions during inter-eruptive periods at Piton de la Fournaise are very low (Di Muro et al. 2016; Tulet et al. 2017; Arellano et al. 2021), unlike the persistent degassing observed at Hawaii (Elias et al. 2018), or other basaltic volcanoes such as Mt. Etna or Stromboli, Italy (Salerno et al. 2009; Burton et al. 2015). Fluctuations in the seismic tremor amplitude can be used as a proxy for gas emission (e.g., Nadeau et al. 2011; Salerno et al. 2018), but can also be affected by other factors including changes in fluid and gas velocities, and the construction and collapse of cones at the eruption site (e.g., Edmonds et al. 2003; Girona et al. 2019). Nonetheless, a fissure opening to allow gas, but not lava, emission generates tremor that can be observed on nearby seismometers (cf. Gottschämmer et al. 2021). At Piton de la Fournaise, the beginning and end of an eruption is declared on the initiation and cessation of seismic tremor (Roult et al. 2012).

A short-lived effusive eruption occurred at Piton de la Fournaise between 2 and 6 April 2020 and ended with an unusual explosive phase (Peltier et al. 2021). Ground-based gas monitoring of the plume was negatively impacted by both the high altitude of the eruptive column and the sub-optimal wind direction (Verdurme et al. 2022), as well as by movement restrictions due to local COVID lockdowns (Peltier et al. 2021). Thus satellite-based observations were crucial for determining the gas emission from the eruption. TROPOMI satellite measurements were combined with the PlumeTraj back trajectory analysis toolkit to derive a sub-daily SO_2_ emission rate time series, which is compared to seismic data, giving a more holistic view of the volcanological processes driving the eruption.

## Piton de la Fournaise

Piton de la Fournaise is the southern of two volcanic edifices making up the island of La Réunion, France (21.244 S, 55.708 E; 2632 m asl). There was a 41-month hiatus, with volcanic activity resuming in June 2014 (Peltier et al. 2016). Between then and April 2020, there were 20 eruptions (Peltier et al. 2021), and a further four between April 2020 and October 2022. The plumbing system of the volcano consists of several magma chambers at a variety of depths, extending from deeper than 15 km almost to the summit (Di Muro et al. 2016; Peltier et al. 2021; Boudoire et al. 2018; Gurioli et al. 2018). The shallowest portion consists of two parts: a shallow sill system (< 2 km below the vent) hosting variably degassed and evolved melt, and a slightly deeper magma chamber (~ 2.5 km below the summit) (Di Muro et al. 2014; Boudoire et al. 2018; Battaglia et al. 2005). The magma residing in the shallow sill system undergoes degassing, cooling, and crystallisation processes, resulting in a degassed magma with H_2_O ≤ 0.8 wt% and S ≤ 1050 ppm (Di Muro et al. 2016). Eruptive activity is driven by regular intrusions of volatile-rich basaltic magma ascending from greater depths, with higher water (1.3–2 wt%) and sulphur (≥ 1600 ppm) contents (Di Muro et al. 2014; 2016).

Piton de la Fournaise has a well-established ground-based monitoring system, managed by the Observatoire Volcanologique du Piton de la Fournaise (OVPF). The system comprises of a network of broadband and short period seismometers, Global Navigation Satellite System (GNSS) receivers, tiltmeters, visible and infrared cameras, weather stations, and gas monitoring stations (Peltier et al. 2022). Gas monitoring includes MAX-DOAS (Multi-Axis Differential Optical Absorption Spectroscopy) instruments operated by the NOVAC network, soil CO_2_ sensors and a MultiGAS station (Peltier et al. 2021; Arellano et al. 2021). The locations of the stations across the island are shown on Fig. 1. The monitoring system has been expanded from five stations in 1981 to 101 in 2020. The MAX-DOAS array, initially installed in 2007, comprises of three stations located 3–4 km from the summit crater, i.e., the Dolomieu (Fig. 1).

During the April 2020 eruption, there were poor weather conditions with low cloud and fog (Fig. S1). The proximal plume was blown to the southwest or west for the first four days of the eruption (Fig. 3, Fig. 4), i.e., away from the DOAS stations that were located to the east of the fissure (Fig. 1). Because La Réunion was under COVID-19 related restrictions during the eruption, all elements of the autonomous monitoring system were particularly important, where the movement of OVPF staff was severely restricted (Peltier et al. 2021). It was therefore impossible to carry out traverse measurements to obtain SO_2_ emission estimates. This coupled with the foggy and cloudy conditions, mean there are no ground-based measurements of SO_2_ flux during the April 2020 eruption (Verdurme et al. 2022).

## Overview of April 2020 eruption

A description of the April 2020 eruption is provided by Peltier et al. (2021) and is summarised here. Precursory activity involved a slow increase in seismicity and ground deformation in the four days before the eruption. A seismic crisis began at 04:15 UTC on 2 April, with shallow volcanic-tectonic (VT) seismicity recorded between 1.5 and 2.5 km beneath the summit and rapid, though small (ca. 10 cm), uplift in the region around the eruptive fissure. Tremor began at 08:20 UTC and a helicopter overflight at ~ 11:00 UTC confirmed the eruption from a fissure on the eastern flank of the volcano. The poor weather conditions meant an eruption plume was not observed on the camera stream until early the next morning at 01:40 UTC (3 April; Fig. S1a and b).

During 2–4 April, activity was characterised by low (< 50 m high) lava fountains aligned along the fissure, along with low frequency tremor. At 02:22 UTC on 5 April, a syn-eruptive VT swarm was observed. Immediately after this swarm, the seismic station closest to the fissure (named ‘FLR’, Fig. 1) was rapidly tilted and then ceased transmission. A helicopter overflight at 06:30 UTC revealed fountain height had increased to exceed 50 m and had become focused at a single crater. Though other volcanoes, such as Kilauea, Hawaii and Mt. Etna, Italy, regularly exhibit lava fountains to hundreds of metres (Parfitt et al. 1995; La Spina et al. 2021), fountains exceeding 50 m at Piton de la Fournaise are unusual (Edwards et al. 2020). Initial reports of Pele’s hair fall occurred during the afternoon of 5 April, with lapilli fallout across most of the island (> 50 km from the vent) continuing until the end of the eruption (Peltier et al. 2021; Verdurme et al. 2022). Acid damage was observed on the Pele’s hair collected on the ground at increasing distances from the eruptive vent (Bourkortt 2021). The final phase of the eruption was also characterised by high lava effusion rates (peaking above 30 m^3^/s), strong high frequency tremor below the Dolomieu cone, edifice inflation, and high CO_2_ fluxes (Peltier et al. 2021).

Tremor abruptly dropped to background levels at 09:30 UTC on 6 April, though a small gas plume was still visible in the cameras at 10:55 UTC (Fig. S1e). This heralded the end of the eruption.

The mean output rate, time-averaged over the whole four-day eruption, was estimated at 16 m^3^/s by Peltier et al. (2021), using HOTVOLC (using geostationary SEVIRI data; https://wwwobs.univ-bpclermont.fr/SO/televolc/hotvolc/) and MIROVA (using polar-orbiting MODIS data; https://www.mirovaweb.it/; Coppola et al. 2016) observations. The time-averaged discharge rates displayed an increasing trend following 5 April, with a maximum value recorded by HOTVOLC of 31.5 m^3^/s at 03:00 UTC on 5 April (Peltier et al. 2021).

## Methods

### TROPOMI

The TROPOspheric Monitoring Instrument (TROPOMI) is flying on board ESA’s Sentinel-5P satellite, launched on 13 October 2017 (Theys et al. 2019). TROPOMI is a polar-orbiting, sun-synchronous UV hyperspectral spectrometer, covering a spectral range of 270–2385 nm. The instrument has a swath width of 2600 km, with a current pixel size of 5.5 × 3.5 km^2^ (improved from 7.0 × 3.5 km^2^ in August 2019). TROPOMI is a heritage UV instrument, following on from the TOMS (Total Ozone Mapping Spectrometer) instruments, first launched in 1978 (Carn et al. 2003), and OMI (Ozone Monitoring Instrument), launched in 2004 (Levelt et al. 2006; McCormick et al. 2013). At the time of writing, OMI is still in operation.

TROPOMI has provided a step change in our ability to measure trace gases from space, especially for point source emissions such as those from volcanoes (e.g. Theys et al. 2019; Burton et al. 2021; Esse et al. in press). The improvement in spatial resolution, from 3° pixels on the first TOMS instrument (McPeters et al. 1993) and 13 × 24 km^2^ pixels on OMI (Levelt et al. 2006), has enabled the measurement of low concentration passive degassing (Queißer et al. 2019) and smaller explosive plumes, such as that produced by the December 2019 eruption of Whakaari/White Island (Burton et al. 2021).

Our analysis used TROPOMI Level 2 Offline SO_2_ data (L2__SO2__, OFFL dataset), obtained from the Copernicus Sentinel-5P Pre-Operations Data Hub (https://s5phub.copernicus.eu/dhus/#/home). The Level 2 data include the Vertical Column Density (VCD) for each pixel. These are produced by converting the Slant Column Density (SCD) (the measurement made at an angle through the atmosphere) into a downward looking observation using an Air Mass Factor (AMF) which incorporates the assumed distribution of SO_2_ within the atmosphere, the satellite viewing geometry, and atmospheric effects. Since the AMF is dependent on the altitude of the gas within the atmosphere, the L2 data are produced using three averaging kernels, each using an AMF with a box profile distribution centred on one of three altitudes (labelled 1, 7, and 15 km, but centred at 0.5 km a.g.l., 7 km a.s.l., and 15 km a.s.l. respectively). A fourth VCD, covering a polluted scene with SO_2_ within a well-mixed boundary layer, is also available but is not used in this analysis. To calculate the true SO_2_ VCD for any given pixel, we linearly interpolate the values for the three layers to the plume altitude. Determining the correct plume altitude is therefore of critical importance in calculating the true mass within a pixel.

### PlumeTraj

PlumeTraj is a back trajectory analysis toolkit designed to calculate the SO_2_ emission history of a SO_2_ plume or cloud from a static satellite UV image. The PlumeTraj methodology is reported in more detail elsewhere (Esse et al. in press; Burton et al. 2021) and so only an overview and key points for this application will be provided here.

For each plume pixel within a satellite image, PlumeTraj calculates the altitude of the plume at the time of measurement (measurement altitude), as well as the time and altitude that the gas in that pixel was emitted (injection time and injection altitude, respectively). Plume pixels are selected using noise and nearest neighbour thresholds applied to the SO_2_ VCD data within a defined region of interest around the injection point (in this case, Piton de la Fournaise volcano). In our case, we used a noise threshold of three times the random VCD error on the pixel and a nearest neighbour threshold which required that at least two of the surrounding eight pixels passed the noise threshold test.

Back trajectories were then calculated for all selected pixels using the pixel centre as the initiation location. To do this, we ran calculations every 100 m over an altitude range of 100–8000 m. The trajectory run time varied from 24 to 60 h to accommodate the changing residence times of the plume on different days (Table 1). All trajectories were computed using the HYSPLIT (Hybrid Single Particle Lagrangian Integrated Trajectory) model (Stein et al. 2015), using the National Oceanic and Atmospheric Administration’s Global Forecast System 0.25° global meteorological data (available from ftp://ftp.arl.noaa.gov/pub/archives/gfs0p25).

**Table 1:** Initiation parameters for the PlumeTraj runs for each day of the eruption

Date	Trajectory run time (hr)	Closest approach threshold (km)	A priori altitude (km a.s.l.)
02/04/20	24	100	3
03/04/20	24	100	3
04/04/20	48	100	3
05/04/20	48	150	3.5
06/04/20	60	200	3
07/04/20	60	250	3
08/04/20	48	250	3

The pixel’s measurement altitude is the initiation altitude for the HYSPLIT trajectory that passes over the injection point. PlumeTraj can use any single point source emission as its injection point, but in our case, the eruption site (21.244 S, 55.708 E) is used. If no trajectories pass directly over the injection point, then the trajectory must pass within a specified distance of the injection point (Table 1). The threshold distance varies from day to day as the threshold encompasses uncertainties in the initial injection conditions as well as instabilities in the model wind fields, which increase with longer analysis times. In the case that more than one trajectory passes over the injection point (as can happen with inversions in the wind field), an a priori solution is determined from direct observations of the remote camera images (Table 1, Fig. S1). The altitude of the optimal trajectory at the point it passes over the injection point is used as the injection altitude. The trajectory run time to this point is subtracted from the measurement time to give the injection time for each pixel.

Uncertainties in the plume altitude are combined with random and systematic errors (included within the TROPOMI L2 data as the precision and trueness respectively) to provide a comprehensive error budget for each pixel, as well as the resulting mass and emission rate time series. The wind field on 6 April was highly divergent in the lower troposphere, with trajectories separated vertically by only 100–200 m producing arrival times that varied by up to 24 h. These large jumps are unlikely to be physically representative of the wind field and so those altitudes producing these unreasonable trajectories were removed, with the analysis performed using those above and below them.

The PlumeTraj results give a pixel-by-pixel plume altitude. These altitudes are used to linearly interpolate between the three VCDs, giving an altitude corrected VCD for each pixel. This is then used to calculate the pixel mass and the overall mass of the plume. By combining the corrected pixel mass with its injection time for all pixels, an SO_2_ emission rate time series can be calculated and related to the mass eruption rate via the injection altitude time series.

### Seismic and camera data

OVPF maintains an array of 24 broadband seismometer and GNSS receiver stations on Piton de la Fournaise (Peltier et al. 2022). A seismic station (FLR, Fig. 1) was destroyed during the eruption, as it was located very close to the eruptive vent. The data presented here are from the next closest station, FJS (Fig. 1). The low frequency tremor data are from the 2–4 Hz band and the high frequency tremor data are from the 8–16 Hz band. Peaks that cross multiple frequency ranges are interpreted as VTs. A detailed description of the seismometer and GNSS network is given in Duputel et al. (2021).

There were also nine visible cameras providing direct observations of the eruption (Fig. S1). These archived data were especially useful due to the lack of near-field observations due to the ongoing COVID-19 lockdown restrictions in place at the time (Peltier et al. 2021). Camera images thus allowed us to assess proximal plume direction and altitude when the local weather conditions permitted.

## Results

**Fig. 2:**
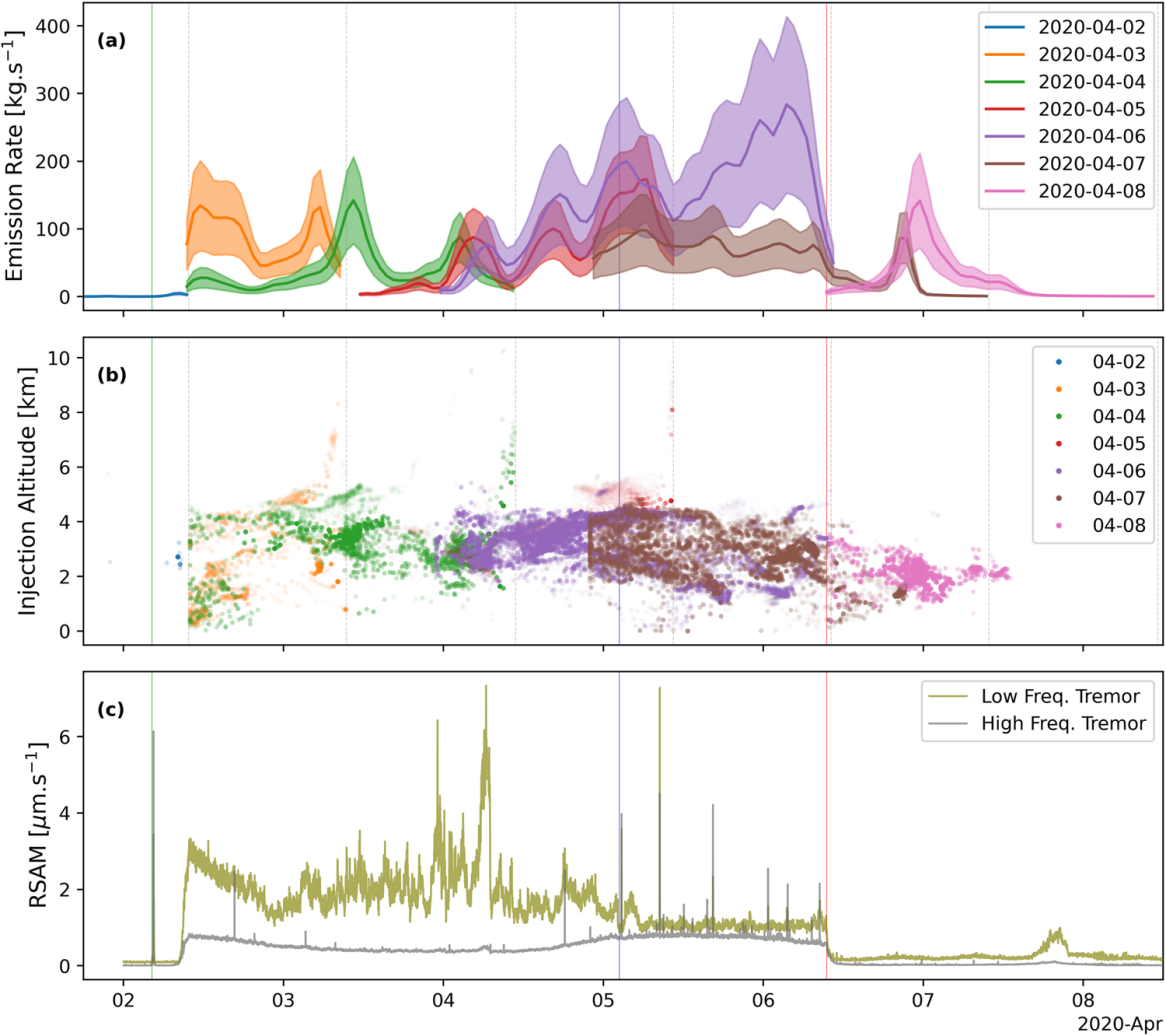
TROPOMI/PlumeTraj-derived (**a**) SO_2_ emission rate; (**b**) injection altitudes (a.s.l.); and (**c**) RSAM (Real-time Seismic Amplitude Measurement) seismic tremor for the eruption period (the spikes are VT events, which cross all frequency bands). Shaded regions in (**a**) give uncertainty bounds on the SO_2_ emission rate. The colour saturation of the point in (**b**) represents the SO_2_ mass of the corresponding pixel (i.e., the darker the colour, the higher the pixel’s mass). Important timings within the eruption are denoted by coloured lines on the panels: green line = start of the eruption; blue line = 5 April event; red line = end of the eruption

**Fig. 3:**
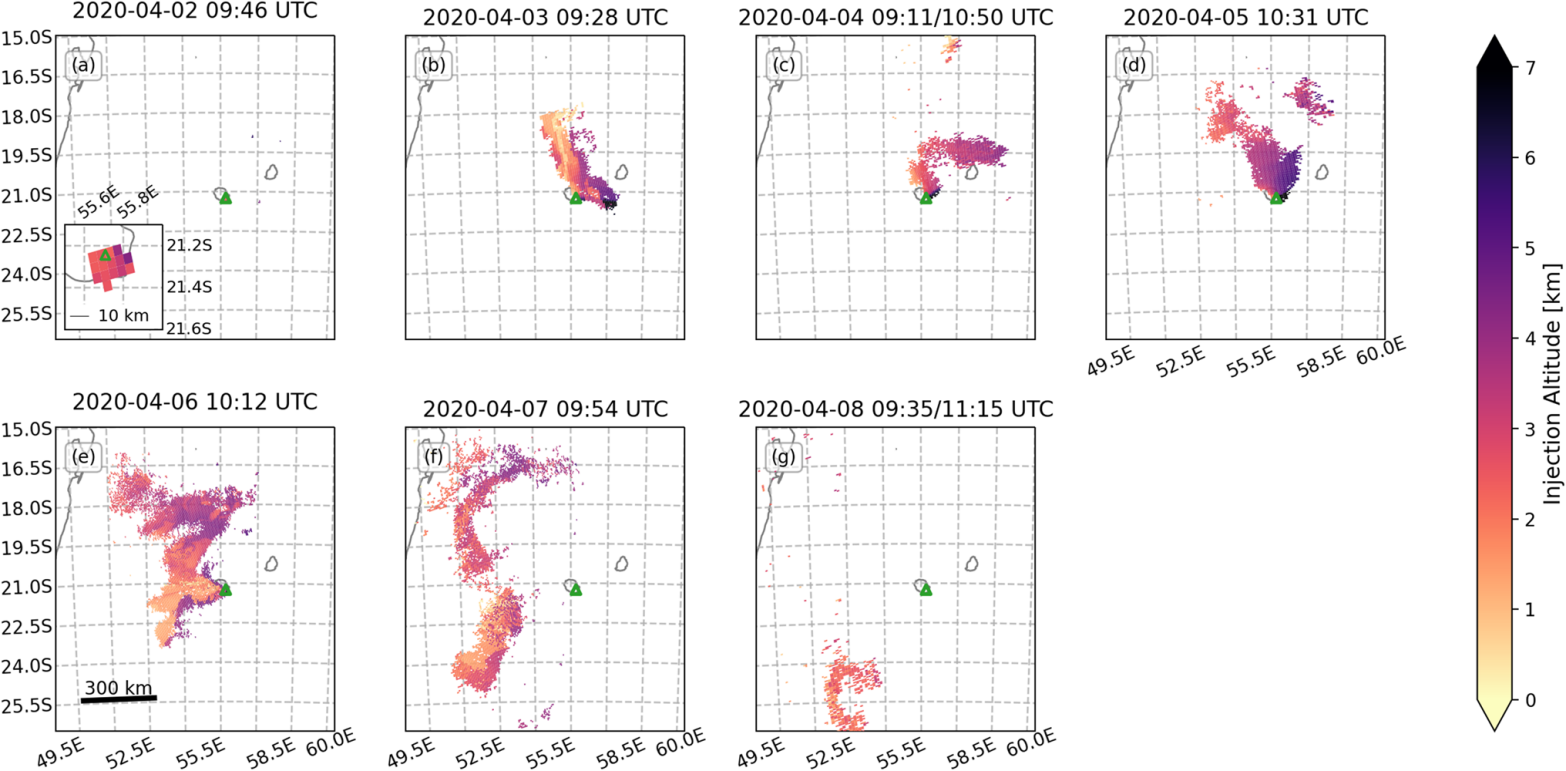
TROPOMI/PlumeTraj-derived injection altitudes for the whole eruptive period. The volcano is denoted by the green triangle. The Cartopy Python library was used to prepare this and all subsequent pixel maps within this paper (Meteorological Office 2015)

**Fig. 4:**
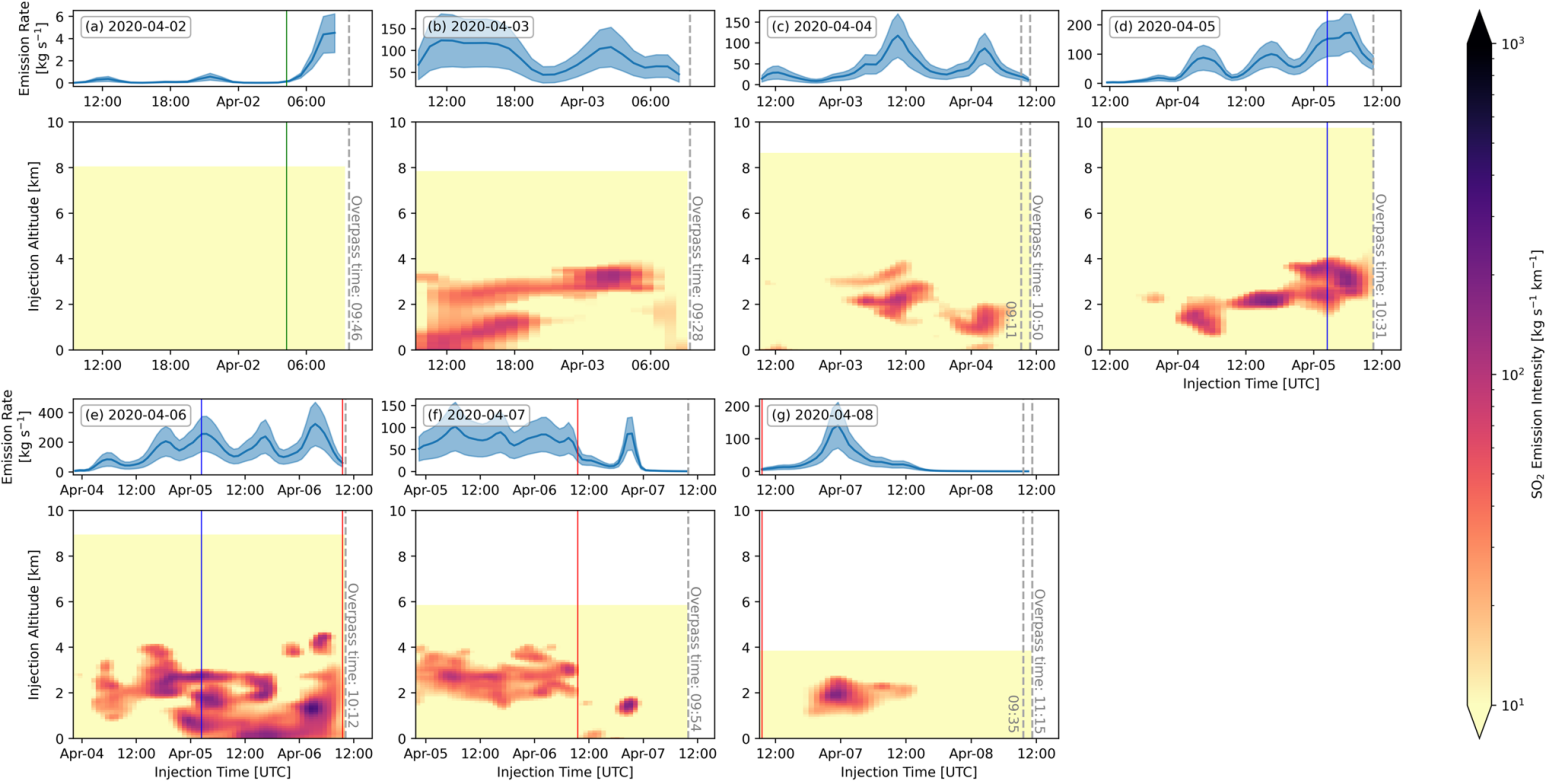
TROPOMI/PlumeTraj SO_2_ emission rate. Important timings within the eruption are denoted by coloured lines on the panels: green line (a, b) = start of the eruption; blue line (d, e) = 5 April event; red line (e, f) = end of the eruption

Low frequency seismic tremor (2–4 Hz) was stronger and more variable in the first three days of the eruption (Fig. 2), prior to the increase in activity on 5 April. Following the increase in activity, the level and variability of low frequency tremor decreased significantly, but high frequency (8–16 Hz) tremor increased (Fig. 2c). There were several VT events beneath the summit during the final two days of the eruption (5–6 April), which is unusual for Piton de la Fournaise (cf. Roult et al. 2012; Duputel et al. 2019). At the end of the eruption, all seismic activity rapidly dropped to background levels (Fig. 2c). PlumeTraj-derived plume injection altitudes were higher (3–4 km, all altitudes are above sea level) before 5 April, peaking at ~ 5 km shortly after the eruptive event of 5 April, and decreasing to 2–4 km thereafter (Figs. 3 and 4).

**Fig. 5:**
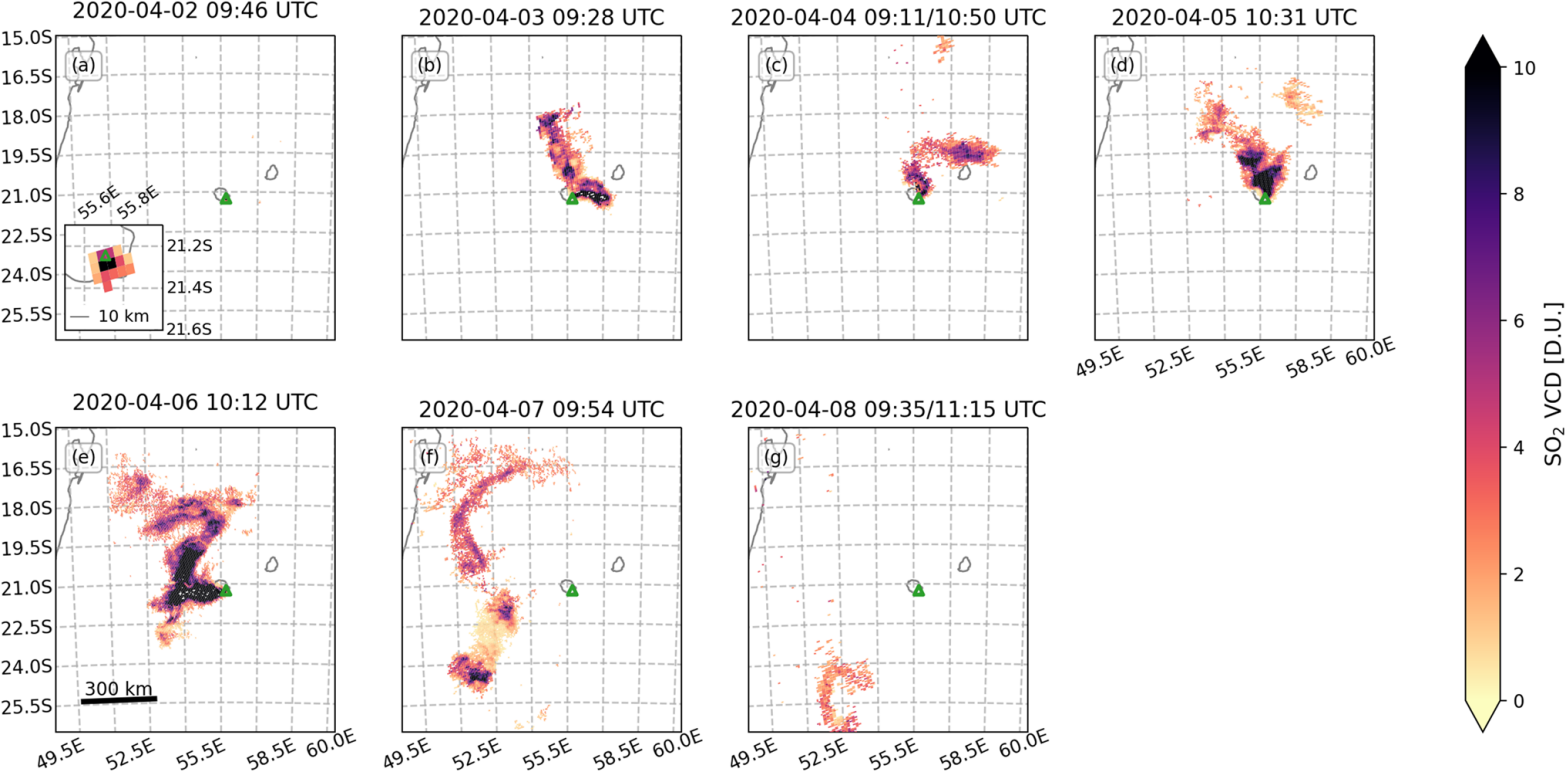
TROPOMI/PlumeTraj-corrected SO_2_ VCD for whole eruption period. The volcano is denoted by the green triangle

The first observation of a plume from TROPOMI on 2 April 2020 was at 09:46 UTC, i.e., 1.5 h after the start the eruption. The plume observed by TROPOMI on this day was small (only 15 pixels, ~ 25 km long by 25 km wide) and was located directly over the eruption site (Figs. 3a, 5a and S2a). The first clear observation of a gas plume associated with the eruption was on 3 April at 09:28 UTC (Figs. 3b and 5b). The plume had initially moved to the north at lower altitudes (1–2 km) before increasing in altitude (3–5 km) and propagating ESE.

The plume on the 4 April was recorded during two orbits (overpass times of 09:11 and 10:50 UTC). The plume initially moved east before the wind direction changed to the north, moving both the previously emitted, easterly plume and the younger plume in a northward direction (Fig. 3c). The altitude of the plume remained approximately constant across both 3 and 4 April, between 3 and 4 km.

A significant change was observed in the hours prior to the event on the 5 April: the plume altitude and emission rate began to increase, with the altitude increasing from 4 km to over 5 km a.s.l. (Figs. 3d and 4d). The plume direction also changed again to move to the north. The altitude of the plume at that time was one of the highest recorded at Piton de la Fournaise (cf. Tulet et al. 2017). The plume altitude then decreased again to 1.5–2.5 km through to the end of the eruption (Figs. 2b, 3e and f and 4e and f).

The eruption ceased a little under an hour before the overpass of 6 April (at 10:12 UTC), but a plume was still visible above the vent and the proximal plume had very young pixel ages (within 10 min of the overpass), indicating continued SO_2_ emission (Figs. 3e and S2e). The pixels to the north of the island were largely the remnant plume from the 5 April, with the emission from 6 April moving to the SW (Fig. 3e). The plume was clearly detached by the overpass of 7 April and had drifted ~ 230 km to the west (Fig. 3f). This remnant plume was substantial, however, with a total mass of 11.3 ± 5.6 kt. This was the first day when the distal plume was significantly impacted by cloud cover, with a visible decrease in the VCDs of the affected pixels (Fig. S3).

Images processed for 7 and 8 April show an SO_2_ emission at ~ 21:00 UTC on 6 April (Figs. 2a and 4e and f), approximately 12 h after the end of the eruption, but there was no corresponding signal seen in the seismicity (Fig. 2c). The total SO_2_ emission from 09:54 on 6 April to 09:54 on 7 April (time of overpass) was 1.7 ± 0.7 kt. The cloud was detected for the final time on 8 April (Fig. 3g), and as on 4 April, the plume was observed over two orbits (09:35 and 11:15 UTC). The northern portion of the 7 April cloud was no longer visible; the SW portion had drifted a further ~ 300 km to the south and contained a total of 4.4 ± 2.1 kt SO_2_.

The trend in the injection altitudes derived from PlumeTraj were corroborated by visible camera images from the Cascades station (CAS, Fig. 1). The cameras do not render absolute altitudes, but the imagery showed higher plumes in the first part of the eruption (Figs. S1a-c), peaking early on 5 April (Fig. S1d), and then decreasing in altitude into 6 April (Fig. S1e). By early on 7 April, no plume was apparent in the camera images (Fig. S1f).

There was high variability across the plume in any given TROPOMI image for both the raw and interpolated VCD data (Fig. 5), with pixels of high and low concentration being in close proximity (i.e., within two or three pixels of each other). This pattern could be explained by a variable emission rate, but also by changes in wind speed and/or direction. However, the PlumeTraj-derived emission times for each pixel (Fig. S2), show that the pattern resulted from highly variable emission rates (Fig. 4).

SO_2_ emission rates for the first 3 days of the eruption (i.e., from 2 to 4 April) were lower, though the rate on 4 April peaked at 142 ± 64 kg/s. A remnant plume was first observed on 4 April, with all following days also producing them (Fig. 4). Consequently, PlumeTraj was run for longer than 24 h for these days (Table 1). The plume altitudes were comparable between the initial result (from the first overpass, when the plume was fresh) and those from the later overpass (when the plume was visible as a remnant). The SO_2_ emission rate began to significantly increase 3–4 h before the eruptive event on 5 April (Figs. 2a and 4d and e, blue line), with a maximum value of 173 ± 63 kg/s being recorded on 5 April. The peak emission rate of the whole eruption was recorded on 6 April and was 284 ± 130 kg/s (Fig. 4e). Lowest plume altitudes of the eruption were also measured on 6 April, as seen in the TROPOMI observations on 6, 7 and 8 April and corroborated by visible camera observations (Fig. S1).

With the end of the eruption on 6 April at 09:28 UTC, the SO_2_ emission did not end as abruptly as the tremor (Fig. 2a and c), however the rate did decrease to around zero in the hours immediately after the end of the eruption (Fig. 4f and g). A pulse of SO_2_ was released late on 6 April and was visible in the results for 7 and 8 April (Fig. 2a). The visible camera also recorded continued emission of a plume for several hours after the end of the eruption.

(Mass and emission rate values for all days between 2 and 8 April, as well as the totals for the whole eruption, can be found in the supplementary material, Table S1.) The total erupted SO_2_ mass, calculated from pixels within 24 h of the overpass on each day, was 34.9 ± 17.4 kt.

## Discussion

The eruption was a tale of two halves:

- The first half (2–4 April) was characterised by largely effusive activity interspersed by very weakly explosive activity (low lava fountains) along a fissure with low frequency tremor and relatively low SO_2_ emission rates;
- The second half (5–6 April) by higher lava fountains, which produced air fall of Pele’s hair, weaker low frequency tremor but stronger high frequency tremor, and higher SO_2_ emission rates.

The wider area covered by Pele’s hair fall, and acid damage to them during the second half of the eruption supports the higher SO_2_ emission rate as obtained from our TROPOMI inversion. Higher SO_2_ concentrations would provide more reactant to form H_2_SO_4_ within the plume, producing the observed acid damage. This also means the SO_2_ mass calculated from TROPOMI will be a lower limit as there must have been rapid chemical conversion within the proximal plume. Pele’s hair fallout during 5 and 6 April (Peltier et al. 2021) correlates with the increase in lava fountaining intensity. Initial pre-eruptive seismicity was a series of VT events located below the summit. Once the eruption began, low frequency tremor increased and remained high, though variable, throughout the first half of the eruption. Following the eruptive event of 5 April, low frequency tremor dropped substantially, and high frequency tremor increased to become the dominant source of seismicity (Fig. 2c). Approximately 100 VT events were also recorded beneath the summit during this period of the eruption.

The initial VT crisis preceded the start of the eruption and is interpreted as magma moving from the upper magma system towards the surface. The seismic crisis, lasting for almost 4 h, was rather long lived compared with normal precursory activity which typically lasts for less than 2 h (Roult et al. 2012). The first part of the eruption (2–4 April) involved the transfer of magma from the shallow (~ 2.5 km b.s.l.) storage zone to the surface. Since the system was connected to the surface and open, seismicity was dominated by tremor related to gas release (e.g. Ripepe et al. 1996; Gottschämmer et al. 2021).

The April 2020 eruption was preceded by an eruption on 10 February 2020. We propose that magma erupted at the beginning of the April 2020 eruption (during 2–4 April) had been resident since at least the February eruption within the very shallow part of the plumbing system. As a result, it was partially degassed, and had a relatively low sulphur content, as seen in the TROPOMI SO_2_ data (Fig. 5a–c). A similar process has been observed during eruptions at other volcanoes such the 2021 eruption of La Soufrière, St. Vincent (Esse et al., in press). An extreme case was the January 1997 eruption from the Napau crater of Kilauea, where no new magma was erupted (Thornber 2001).

Bulk rock analysis performed by the DynVolc Observation System (DynVolc 2013) showed MgO magma content increased from 7.2 wt% at the beginning of the eruption to 9.8 wt% on the 5 April and then decreased slightly (Table S2). The most magnesian magmas were erupted after the 5 April eruptive event and contained a higher amount of olivine but also had a higher alkalinity index and different ratios of highly incompatible trace elements (higher Nb/U, lower Th/Yb ratios) with respect to the magma erupted prior to 5 April. That suggests that the more mafic magma represented the new magma input that ‘pushed out’ the system-stored degassed magma. The variability in the gas emission rate (Figs. 2a and 4) and tremor (Fig. 2c) are thus both related to the evacuation of magma arising from variably degassed sources within the shallow crustal plumbing system (Di Muro et al. 2014; Verdurme et al. 2022).

**Fig. 6:**
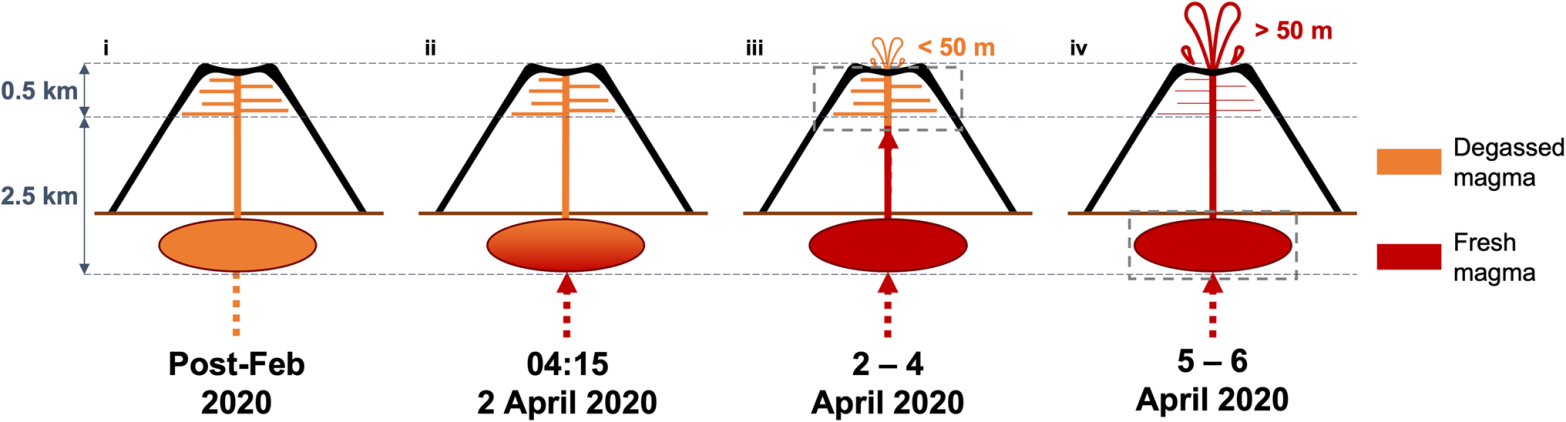
Schematic of the upper plumbing system at Piton de la Fournaise, denoting the different stages of the eruption and the portions of the system being accessed at each stage. Orange = degassed magma, red = fresh, more mafic magma. (i) Between the February and April 2020 eruptions, magma remained in the shallow sill system, degassing; (ii) a fresh injection of mafic magma at depth led to the start of the April eruption, as seen in the seismic crisis; (iii) from 2 to 4 April, the older, degassed resident magma was pushed out by the fresh, new magma, low lava fountains (up to 50 m) were observed, along with a low SO_2_ emission rate; (iv) after the eruptive event on 5 April, the fresh magma began to be erupted, the sill system had been evacuated, there were higher lava fountains (> 50 m), and the SO_2_ emission rate was much higher. The schematic is not to scale.

The seismicity observed during and following the eruptive event of 5 April, i.e., high frequency tremor and syn-eruptive VTs, is uncommon at Piton de la Fournaise. Most of the events were located 0.2–0.8 km a.s.l., at the top of the shallowest magma reservoir. This suggests instability in the summit region possibly caused by the transfer of magma within the uppermost part of the plumbing system as it was emptied and partially refilled by the arrival of new magma. We propose that as the eruption progressed and magma continued to erupt from the uppermost part of the plumbing system, the overpressure of the whole system decreased (cf. Coppola et al. 2017). The arrival of the deeper mafic magma in the upper portion of the plumbing system led to the change in the eruption intensity and increase in gas emissions on 5 April. We interpret the VT events at this time as movement along faults in the country rock during upward magma migration. This more mafic magma was hotter and more volatile rich than the magma already resident in the shallow system, explaining the increase in SO_2_ emission rate observed in the latter part of the eruption (Fig. 5d–f) and the higher lava fountains. The eruption of more mafic magmas and increased TADR during the final phase of an eruption at Piton de la Fournaise has been documented in the past during long lasting eruptions, such as that of August 2015 (Coppola et al. 2017; Sundermayer et al. 2020), and is associated with important shifts in eruptive behaviour (Verdurme et al. 2022). Gas passage was more difficult in the initial phase of the April 2020 eruption, passing through more evolved magma in the upper storage system. Once this plug of viscous magma had been extruded, the gas was able to pass through the ascending magma column more easily. The weaker but steady low frequency tremor measured during the second phase suggests the system was approaching a steadier state, with the gas being emitted in a relatively continuous way. Once this fresh batch of magma was erupted, the driving force was exhausted, and the eruption ended (Fig. 6).

Cloud cover is a significant issue for satellite-based remote sensing in the UV and IR. In our case, the main SO_2_ cloud was relatively unaffected for the first five days (though the distal plume was not impacted by cloud, there was local cloud cover directly over the island, as discussed previously). The distal plume on 7 April was affected by cloud cover to the SW of La Réunion (Figs. 3f and S3f), with pixels having a lower VCD than those surrounding the area covered by cloud. This is a result of the plume being below a layer of semi-transparent cloud, which absorbed some of the signal. The SO_2_ emission rate (Fig. 2a) shows this effect, with a lower flux than expected from 24 to 36 h before the observation of 7 April. This means the total SO_2_ mass derived from the image of 7 April was an underestimate. However, since the pixels impacted had ages mainly > 24 h, it did not affect the SO_2_ mass estimated for the entire eruption, as this only included pixels with ages of < 24 h.

Plume injection altitudes from PlumeTraj agree with camera observations, with a higher plume in the first part of the eruption, peaking during the eruptive event of 5 April, and then decreasing following 5 April. The SO_2_ plumes were regularly seen to have changed direction (Figs. 3 and 5). This could have been caused either by changes in the injection altitude or in local wind patterns. Since PlumeTraj uses wind fields initiated every 3 h, it is possible to resolve the impact of each of these processes and return an injection altitude which also accounts for the perturbations caused by local weather patterns after emission.

Some trajectories, especially those of 6 April, produced injection altitudes slightly lower than that of the vent (i.e., minimum altitudes were ~ 1.5 km compared with a vent elevation of ~ 1.95 km). In the case of a weak emission, it would be possible for the plume to be entrained into the ambient wind field and ‘dragged’ down the flank of the volcano, resulting in a lower apparent injection altitude than if there had been no topography present. However, this solution is not supported by the visible camera images (Fig. S2). An alternative explanation is that since the wind model has a resolution of 0.25°, the full elevation range over the volcano will not be entirely captured, which can lead to small altitude discrepancies for trajectories close to ground level. Instability in the model within the lowest region of the atmosphere was seen in the analysis for 6 April. A very strong wind shear, with similar directions but very different wind speeds, led to differences in arrival times of over 24 h for trajectories separated by only 100–200 m. A limited number of trajectories, with large differences in arrival time to the trajectories adjacent, were thus discarded from the analysis.

Our results show an increase in the SO_2_ emission rate late on 6 April. The increase was not accompanied by seismicity and so appears to have been an aseismic release of SO_2_ following the end of the eruption. Due to the lockdown restrictions, we have no visible corroboration of this emission, but the results from both 7 and 8 April show this injection (Fig. 2a and c). Given the instability in the wind field on 6 April, an alternative explanation is that this is an artefact in the model propagated through the subsequent days.

As seen in Fig. 4, SO_2_ plumes observed by TROPOMI were erupted over more than the 24 h prior to the overpass. If all pixels were assumed to have been erupted within the 24 h before the measurement, this could result in a substantial mass overestimate due to double counting if the image includes gas that is more than 24 h old. Several techniques have been developed to address this issue (e.g., Bluth and Carn 2008; Krotkov et al. 2010; Theys et al. 2013). The mass from the previous day could be subtracted from the new measurement, but this could potentially lead to an underestimate of the emitted mass if the plume is resident for less than 24 h. The Delta-M method of Theys et al. (2013) was developed by to take into account plume lifetime issues, but requires the user to know, or be able to calculate, an SO_2_ loss rate. Finally, an arbitrary physical cut off could be applied, with the user choosing which areas of the plume to include in any mass calculation. All these methods have drawbacks and were often labour-intensive and involved a significant amount of interpretation. They also only provide a single daily average measurement. Instead PlumeTraj produces sub-daily emission rates. Using the PlumeTraj results, we can establish which pixels contained SO_2_ emitted in the 24 h prior to each overpass. These can then be extracted and summed to give a daily mass. The cumulation of each daily value then gives the total erupted SO_2_ mass for the eruption, which was 34.9 ± 17.4 kt.

There are a variety of sinks for SO_2_ in the atmosphere including chemical conversion (into sulphate), wet and dry deposition, and diffusion (e.g., Bluth and Carn 2008; Carn et al. 2016; Lachatre et al. 2022), all of which can reduce concentrations in a pixel to below TROPOMI’s detection limit. PlumeTraj is only able to consider the SO_2_ remaining in the atmosphere when the image is acquired. HYSPLIT, as with many trajectory models, does have the ability to include a loss term, but this functionality is only available when running trajectories in forward mode, not in reverse. There is thus no way to include gas that we are no longer able to see in the image due to loss. However, it does mean that there is an unrepresented loss term in our results, so they must be considered a lower limit.

Verdurme et al. (2022) used a single plume altitude to calculate the SO_2_ mass seen by TROPOMI (and other satellite instruments), giving a total SO_2_ mass of 17.32 ± 5.20 kt (using an estimated error of 30%). This is lower than our calculated value of 34.9 ± 17.4 kt but is within error; the discrepancy showing the importance of using the correct plume altitude in the mass calculation. Verdurme et al. (2022) also calculated a magmatic sulphur content of ~ 2000 ppm from the TROPOMI data (assuming the complete release of the pre-eruptive sulphur as measured in melt inclusions), which would suggest the involvement of a deeper magma reservoir. This agrees with our assertion that ascent of a more mafic and volatile-rich magma into the upper part of the plumbing system drove the increase in intensity of the eruption on 5 April, and that the eruption ended when this fresh material had been exhausted (Fig. 6).

## Conclusion

The April 2020 eruption of Piton de la Fournaise was an unusually intense event lasting ~ 4 days and releasing 34.9 ± 17.4 kt of SO_2_. The eruption had two phases: the first (2 through 4 April) characterised by relatively low SO_2_ emission rates (~ 50–140 kg/s), strong low frequency tremor and low fountains. This was followed by an eruptive event in the early hours of 5 April 2020, leading to the second phase (5–6 April, when the eruption ended), which included higher lava fountaining, significantly higher SO_2_ emission rates, increased high frequency, and decreased low frequency, tremor. The plume injection altitude also peaked during the 5 April eruptive event at ~ 5 km a.s.l. Using the PlumeTraj toolkit, the SO_2_ emission rate was found to peak at 284 ± 130 kg/s on 6 April. The 5 April eruptive event is attributed to a change in magma supply to the shallow system, with an additional injection of a hotter, volatile-rich magma which led to the increased SO_2_ emission rate, fire fountain heights, and widespread air fall of Pele’s hair. The injection altitude, SO_2_ emission rate, and local meteorological conditions were all dynamic and varied throughout the eruption. This complexity highlights the importance of performing the back trajectory analysis on each pixel within a satellite image, as this approach allows the detailed evolution of the gas emission to be investigated. The application of the PlumeTraj toolkit enabled remnant SO_2_ from previous days to be separated from the daily cumulative SO_2_ masses and refines, but overlaps with, the estimations performed by Verdurme et al. (2022), allowing us to reach similar conclusions. That is, the increase in intensity at the end of the eruption resulted from the arrival of fresh magma.

The April 2020 eruption of Piton de la Fournaise represents an example of decoupling between the intensity of low frequency seismic tremor and the intensity of magma and gas emission rates. The low frequency tremor intensity was at its peak during the initial phase of the eruption when magma and SO_2_ emissions were low. However, low frequency tremor decreased during the second phase when magma and gas emissions were higher. The emptying of magma stored in the upper portion of the plumbing system in the first part of the eruption, and the injection of deeper, gas-rich magma during the second half of the eruption led to strong high frequency tremor and syn-eruptive VT events, as well as an increase in SO_2_ and lava emission rates. This suggests that the observation of high frequency tremor may be diagnostic of the paroxysmic phase of an eruption of Piton de la Fournaise.

Our study shows the importance of combining multiple data sets when monitoring volcanoes. The seismic and gas data sets alone would have led to an incomplete, and likely incorrect, interpretation of the eruption and system dynamics that fed it. By combining seismic and gas data sets, a well-constrained and accurate assessment of the system dynamics driving the eruption can be made and changes in activity revealed, leading to improved risk assessments.

## Supplementary Information

Below is the link to the electronic supplementary material.

Supplementary file1 (PDF 4005 KB)

## Data Availability

All results data used in this study are stored as NetCDF files in the following repository: 10.6084/m9.figshare.20439873. The Level 2 TROPOMI OFFL data are available from the Sentinel-5P Pre-Operations data hub (https://s5phub.copernicus.eu/dhus/#/home). All OVPF seismic data are available from http://resif.fr/.
