# Supplementary material for: Unusually high SO2 emissions and plume height from Piton de la Fournaise volcano during the April 2020 eruption
Source: Bull Volcanol. 2023 Mar 8;85(4):21. doi: 10.1007/s00445-023-01628-1 (PMC9993386; doi:10.1007/s00445-023-01628-1)
Supplement: Supplementary file 1 — Supplementary file1 (PDF 4005 KB) [file 445_2023_1628_MOESM1_ESM.pdf]

Supplementary Material for "SO_2 emissions and tremor evolution from Piton de la Fournaise volcano during the April 2020 eruption", Bulletin of Volcanology

Authors: C. Hayer*[1], M. Burton[1], V. Ferrazzini[2,3], B. Esse[1], A. Di Muro[2,3]

* Corresponding author (email: catherine.hayer@manchester.ac.uk)

Table S1 *TROPOMI/PlumeTraj-derived total SO_2 mass and peak emission flux for the whole eruptive period*

Date	Mass (kt)		Peak flux 24 hr (kg/s)
	All pixels	24 hr	
02/04/2020	0.061 ± 0.025	0.061 ± 0.025	4.7 ± 1.8
03/04/2020	7.6 ± 3.4	7.6 ± 3.4	135 ± 66
04/04/2020	7.1 ± 3.3	4.4 ± 1.9	142 ± 64
05/04/2020	11.4 ± 4.8	8.8 ± 3.5	173 ± 63
06/04/2020	30.5 ± 14.4	16.6 ± 7.7	284 ± 130
07/04/2020	11.3 ± 5.6	1.7 ± 0.7	87 ± 38
08/04/2020	4.4 ± 2.1	0.4 ± 0.2	22 ± 11
**Total**	**72.2 ± 33.7**	**34.9 ± 17.4**	

Table S2 *Whole rock chemical analysis for samples taken throughout the April 2020 eruption*

Date	February 2020	February 2020	February 2020	April 2020	April 2020	April 2020	April 2020
Sample Reference	REU 200213-1	REU 200213-2	REU 200228-1	REU 200511-1	REU 200511-2	REU 200505-3	REU 200409-6
Collection date	13/02/20	13/02/20	10/02/20	02/04/20	02/04/20	05/04/20	06/04/20
**Major elements (from ICP-AES, LMV)**							
SiO_2	47.46	48.82	48.74	48.82	48.53	47.95	48.85
Al_2O_3	13.67	13.47	13.42	13.86	13.73	12.82	13.00
FeO(t)	11.25	11.60	11.20	11.35	11.24	11.63	11.23
MnO	0.18	0.18	0.18	0.18	0.18	0.18	0.18
MgO	8.35	8.48	7.73	7.15	7.16	9.68	8.03
CaO	11.99	11.27	11.70	11.65	11.67	10.40	10.81
Na_2O	2.48	2.56	2.20	2.86	2.84	2.81	2.97
K_2O	0.61	0.68	0.75	0.79	0.73	0.73	0.76
TiO_2	2.60	2.69	2.68	2.79	2.77	2.59	2.64
P_2O_5	0.35	0.35	0.34	0.38	0.38	0.33	0.34
**Total**	**98.94**	**100.11**	**98.94**	**99.83**	**99.22**	**99.13**	**98.79**
**Trace elements (from ICP-MS, IPGP)**							**(from CP-MS, Vlastelic)**
Nb/Ta	17.38	17.96	16.78	16.37	15.35	16.12	16.29
Nb/U	47.93	46.28	45.34	44.34	45.26	46.60	40.57
Th/Ta	1.41	1.50	1.38	1.25	1.28	1.33	1.55
Th/Nd	0.07	0.07	0.07	0.07	0.07	0.07	0.08
Th/Sm	0.28	0.28	0.27	0.27	0.28	0.27	0.31
Th/Yb	0.80	0.80	0.80	0.83	0.83	0.79	0.87
Th/U	3.90	3.85	3.72	3.38	3.77	3.83	3.85
Th/La	0.10	0.10	0.10	0.09	0.10	0.10	0.11
Ba/Sr	0.32	0.34	0.34	0.31	0.30	0.30	0.35
Rb/Sr	0.04	0.04	0.04	0.04	0.04	0.04	0.05
La/Sm	2.89	2.88	2.77	2.83	2.87	2.79	2.88
La/Yb	8.34	8.25	8.36	8.81	8.50	8.21	7.99
Ni/Sc	4.70	4.49	3.32	3.33	2.98	7.02	4.21
Eu*	0.58	0.58	0.57	0.57	0.57	0.56	0.56

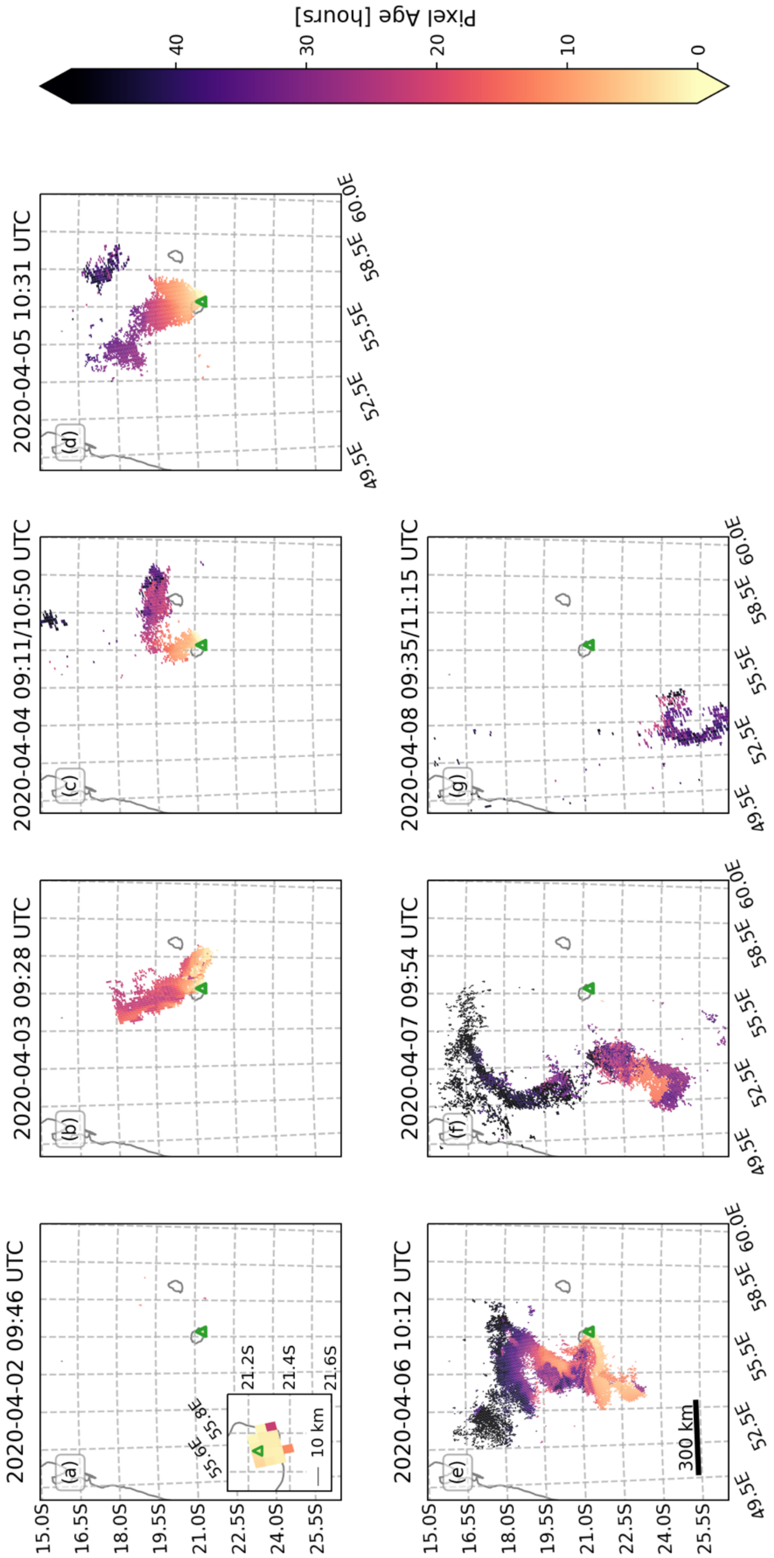

Fig. S1 *TROPOMI/PlumeTraj-derived pixel age for the whole eruptive period. The volcano is denoted by the green triangle. The age is from the overpass time in the title of each panel*

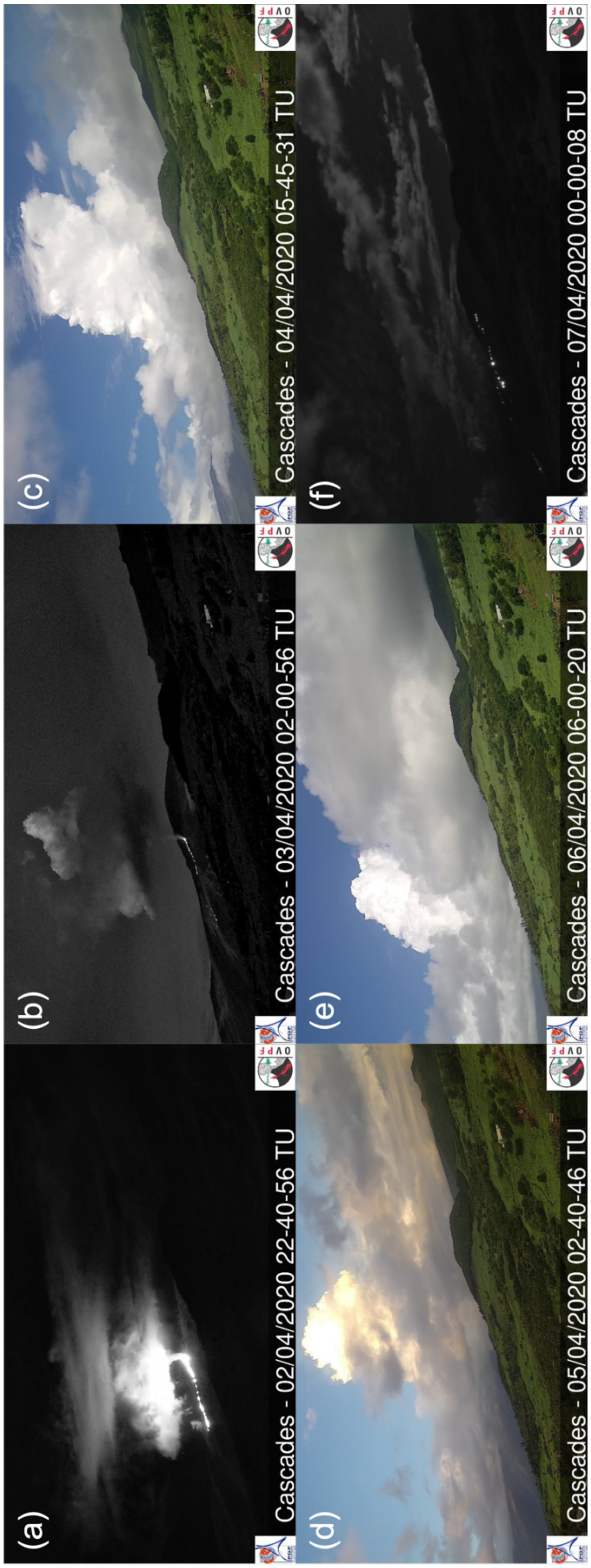

Fig. S2 *Images from visible and infrared cameras at the Cascades station, from the whole eruption period, showing the impact of meteorological cloud and plume height decreases at the end of the eruption. The relative location of the station is shown in Fig. 1 (CAS)*

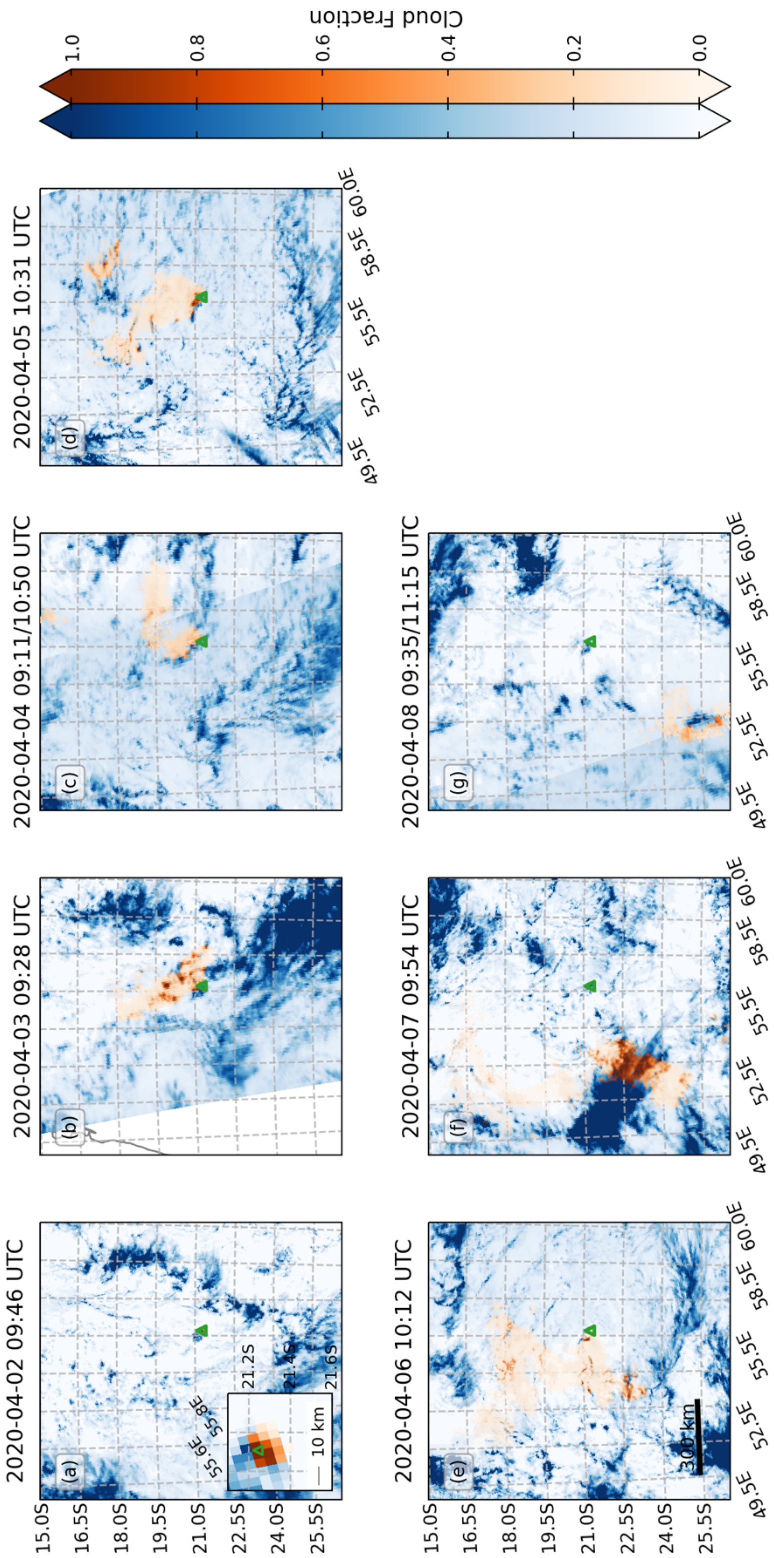

Fig. S3 *TROPOMI cloud fraction per pixel for the whole eruptive period. The orange colour map refers to pixels within the plume; the blue colour map refers to pixels outside of the plume. The plume is largely unaffected by cloud cover, except for a portion of the plume on the 7 April (f) and potentially on the 8 April (g)*
